# Estimating and comparing incidence and prevalence of chronic diseases by combining GP registry data: the role of uncertainty

**DOI:** 10.1186/1471-2458-11-163

**Published:** 2011-03-15

**Authors:** Pieter H van Baal, Peter M Engelfriet, Rudolf T Hoogenveen, Marinus J Poos, Catharina van den Dungen, Hendriek C Boshuizen

**Affiliations:** 1 Expertise Centre for Methodology and Information Services, National Institute for Public Health and the Environment Antonie van Leeuwenhoeklaan, 9 Bilthoven, 3720 BA, The Netherlands; 2 Institute of Health Policy & Management/institute for Medical Technology Assessment, Erasmus University, Rotterdam, Burgemeester Oudlaan, 50 Rotterdam, 3000 DR, The Netherlands; 3 Centre for Prevention and Health Services Research, National Institute for Public Health and the Environment, Antonie van Leeuwenhoeklaan, 9 Bilthoven, 3720 BA, The Netherlands; 4 Centre for Public Health Forecasting, National Institute for Public Health and the Environment, Antonie van Leeuwenhoeklaan, 9 Bilthoven, 3720 BA, The Netherlands

**Keywords:** incidence, prevalence, Monte Carlo simulation, uncertainty

## Abstract

**Background:**

Estimates of disease incidence and prevalence are core indicators of public health. The manner in which these indicators stand out against each other provide guidance as to which diseases are most common and what health problems deserve priority. Our aim was to investigate how routinely collected data from different general practitioner registration networks (GPRNs) can be combined to estimate incidence and prevalence of chronic diseases and to explore the role of uncertainty when comparing diseases.

**Methods:**

Incidence and prevalence counts, specified by gender and age, of 18 chronic diseases from 5 GPRNs in the Netherlands from the year 2007 were used as input. Generalized linear mixed models were fitted with the GPRN identifier acting as random intercept, and age and gender as explanatory variables. Using predictions of the regression models we estimated the incidence and prevalence for 18 chronic diseases and calculated a stochastic ranking of diseases in terms of incidence and prevalence per 1,000.

**Results:**

Incidence was highest for coronary heart disease and prevalence was highest for diabetes if we looked at the point estimates. The between GPRN variance in general was higher for incidence than for prevalence. Since uncertainty intervals were wide for some diseases and overlapped, the ranking of diseases was subject to uncertainty. For incidence shifts in rank of up to twelve positions were observed. For prevalence, most diseases shifted maximally three or four places in rank.

**Conclusion:**

Estimates of incidence and prevalence can be obtained by combining data from GPRNs. Uncertainty in the estimates of absolute figures may lead to different rankings of diseases and, hence, should be taken into consideration when comparing disease incidences and prevalences.

## Background

Morbidity rates, such as disease incidence (number of new disease cases within a certain period) and disease prevalence (current stock of diseased at a certain point in time), are core indicators of public health and health care needs of a population. Furthermore, incidence and prevalence rates are also crucial inputs for burden of disease studies [1-4] and of simulation models designed to making projections of future population health [5-8]. In all these cases, a comparative perspective is implicitly or explicitly chosen: the incidence and prevalence figures of diseases relative to each other count as much as these absolute figures themselves. The manner in which the various morbidity rates stand out against each other provide guidance as to which diseases are most common and what health problems deserve priority. It is not surprising that comparisons of diseases in terms of incidence or prevalence are frequently made by disease advocates, pharmaceutical companies, politicians and journalists.

Obtaining the necessary data to produce comprehensive estimates of incidence and prevalence on a systematic and regular basis is a major obstacle. As a result, for a lot diseases incidence or prevalence are estimated indirectly [9-14]. Even when suitable sources to estimate incidence and prevalence are available, the estimates obtained from them will be subject to uncertainty. Uncertainty surrounding point estimates of population incidence and prevalence may be due to limitations of sample sizes, differences in diagnostic criteria, and methods of case finding. Moreover, the impact of such differences may have different consequences for different diseases. Some sort of quantitative insight into the magnitude of the uncertainty of the estimates is essential when interpreting the results. This should also be borne in mind when policy makers and epidemiologists rank chronic diseases in terms of incidence and prevalence when formulating health policies. Uncertainty in estimates of incidence and prevalence may lead to different orderings of diseases in a 'league table or ranking' when confidence intervals of different diseases overlap. As these rankings are actually used in practice it is important to appreciate the impact of such uncertainty margins on the hierarchical ordering of diseases.

In this study we demonstrate how routinely collected data from general practice registration networks (GPRNs) can be used to estimate incidence and prevalence figures for the most common chronic diseases. Furthermore, we also focus on the role of uncertainty. In the Netherlands, when patients seek medical care from a specialist they have to be referred by their GP, and after consultation the medical specialist reports back to the patient's GP. As a result, GPs have contact with patients suffering from diseases in various stages of their disease and with all patient groups without selection regarding age, gender, socio-economic status or ethnicity [15-18]. We will focus on the role of uncertainty by presenting different outcomes. First, we will present confidence intervals surrounding point estimates of incidence and prevalence. In doing so, we distinguish different levels of uncertainty assessed with multilevel regression modelling. Next, we will illustrate how uncertainty in the estimates affects the ranking of the various diseases in a "league table of morbidity", i.e. a list of diseases in descending order of their prevalences (or incidences). Taking into account uncertainty, turns a league table into a "stochastic" league table. Presenting outcome measures in terms of stochastic league tables is common in economic evaluations [19,20], but has not yet been applied to the ranking of diseases.

## Methodology

### Data

The data used for this study was collected during 2009 for the Dutch Public Health Forecasts Report that is published every 4 years [21]. That report describes public health on the basis of core indicators, including incidence and prevalence for a selection of more than 50 diseases. In this paper we will present estimates of incidences for the year 2007 and prevalences at 1 January 2007 of a subset of 18 chronic diseases using data from five different GPRNs which are non-overlapping in terms of GP practices:

• CMR (Continuous Morbidity Registry Nijmegen): this registration, which started in 1971, consists of 4 GP practices (11 GPs) delivering service to 12,000 persons located in the eastern part of the Netherlands;

• LINH (Netherlands Information Network of General Practice): this registration, which started in 1991, consists of 80 GP practices (160 GPs) delivering service to 350,000 persons. GP practices are located all over the Netherlands;

• RNH (Registration Network General Practices): this registration, which started in 1988, consists of 22 GP practices (65 GPs) delivering service to 88,000 persons located in the southern part of the Netherlands;

• RNUH-LEO (Registration Network of General Practitioners Associated with Leiden University): this registration, which started in 1989, consists of 4 GP practices (20 GPs) delivering service to 30,000 persons located in the Western part of the Netherlands;

• Transitie (Transition Project): this registration, which started in 1985, consists of 5 GP practices (8 GPs) delivering service to 13,000 persons located in the Northern part of the Netherlands.

Approval to use the data for the Dutch Public Health Forecasts Report and related publications was obtained from each GPRN (the data are anonymous and aggregated, requiring no ethics approval, is not publicly available).

When using data from GPRNs for the estimation of incidence and prevalence it is important to realize the differences among GPRNs [22]. Two types of differences are especially relevant. First, several classification systems are in use: three of the five GPRNs use ICPC-1 (International Classification of Primary Care 1) to classify diseases, the Transition project uses ICPC-2 and CMR-N uses E-codes [22]. To account for these differences in classification, we mapped out the codes between these registries, which in some cases required combining codes. For example, the code for diabetes in ICPC is T90, which corresponds with E-codes 0910, 0911 and 0919. Second, methods of data collection are episode-based in some registries, but problem-based in others. Problem-based data contain only information about health problems that are permanent, chronic (duration longer than 6 months) or recurrent. Episode-based data have information about all health problems, at least when a person seeks medical care. For the analyses in this paper we only used incidence and prevalence counts using a classification such that each person can only be an incident case once, and that prevalence is defined as anyone in the registration who has been diagnosed previous to 1 January 2007. Table 1 displays the diseases selected for our exercise, the registrations for which we had data on prevalence and incidence counts for those diseases and the ICPC-1 and E-codes we used to classify cases (ICPC-2 codes were mapped into ICPC-1 codes).

**Table 1:** selection of diseases

Disease name	General practitioner registration networks for which incidence data was available	General practitioner registration networks for which incidence data was available	E-codes	ICPC1-codes	Age range included in the analyses
Diabetes	All 5	All 5	*0910*	*T90*	0-4, 5-9, etc., 85+

Heart failure	All 5	All 5	*2131*	*K77*	50-54, 55-59, etc., 85+

COPD	All 5	All 5	*2480*	*R91+R95*	30-34, 35-39, etc., 85+

Stroke	All 5	CMR/RNH/RNUH-LEO	*1559*	K90	25-29, 30-34, etc., 85+

Coronary heart disease	All 5	CMR/RNH/RNUH-LEO	*2110+2120*	K74+K75+K76	25-29, 30-34, etc., 85+

Dementia	All 5	CMR/RNH/RNUH-LEO/Transitie	*1270*	P70	50-54, 55-59, etc., 85+

Schizophrenia	All 5	CMR/RNH/RNUH-LEO Transitie	*1250*	P72	10-14, 15-19, etc., 85+

Parkinson	All 5	All 5	*1570*	*N87*	30-34, 35-39, etc., 85+

Multiple sclerosis	All 5	All 5	*1560*	*N86*	10-14, 15-19, etc., 85+

Epilepsy	CMR/RNH/RNUH-LEO/Transitie	CMR/RNH/RNUH-LEO/Transitie	1580	N88	0-4, 5-9, etc., 85+

Macular degeneration	Transitie/RNH/RNUH-LEO	Transitie/RNH/RNUH-LEO		F84	30-34, 35-39, etc., 85+

Glaucoma	CMR/Transitie/RNH/RNUH-LEO	CMR/Transitie/RNH/RNUH-LEO	1800	F93	25-29, 30-34, etc., 85+

Cataracts	CMR/Transitie/RNH	CMR//RNH	1790	F92	30-34, 35-39, etc., 85+

Hearing impairments	CMR/Transitie/RNH/RNUH-LEO	CMR/RNH	1890	H84+H85+H86	0-4, 5-9, etc., 85+

Inflammatory bowel diseases	All 5	CMR/Transitie/RNUH-LEO	*2852*	D94	0-4, 5-9, etc., 85+

Osteoporosis	CMR/Transitie/RNUH-LEO/LINH	CMR/Transitie/RNUH-LEO	4154	L95	25-29, 30-34, etc., 85+

Rheumatoid arthritis	CMR/RNH/RNUH-LEO/LINH.	CMR/RNH/RNUH-LEO	4050	L88	0-4, 5-9, etc., 85+

Arthritis	CMR/RNH/LINH/RNUH-LEO	RNH/RNUH-LEO	4061 + 4062 + 4069	L89 + L90 + L91	30-34, 35-39, etc., 85+

CMR: Continuous Morbidity Registry Nijmegen; LINH: Netherlands Information Network of General Practice; RNH: Registration Network General Practices; RNUH-LEO: Registration Network of General Practitioners Associated with Leiden university; Transitie: Transition Project

Furthermore, for each of these 5 registrations the following data were available: population size, as of January 1 2007; numbers of person years (the years lived by the total population in the GP registration during the registration period) for 2007. All data were grouped by sex and 18 age classes (0-4, 5-9, etc., 85+). For diseases that are only relevant at old age we discarded lower age categories.

### Regression modelling strategy

To capture the hierarchical structure of the data incidence and prevalence were estimated using generalized linear mixed models [23]. The possible systematic differences between the GPRNs registrations were taken into account by including a GPRN identifier as a random intercept. The random intercept in our model is meant to capture all of the differences that exist between GPRNs that may be the result of a range of different underlying reasons (differences in case definition, differences in socio-economic status, differences in GP practices, differences in GP computer software).

Incidence was estimated assuming a Poisson distribution and using a log link function. Prevalence was estimated using a logit link function and a binomial distribution for the outcome variable. Age and sex were the only determinants ("fixed effects") included in our dataset, so incidence and prevalence were estimated as a function of polynomials of age, sex and interactions between these variables. To reduce the risk of multi-collinearity we used orthogonal polynomials of age as explanatory variables. To select the 'optimal' model in terms of highest order polynomials in age and the number of interactions between those polynomials and sex we used the Bayesian Information Criterion [24]]. Using a criterion based approach to select an 'optimal' model circumvents the problems associated with a sequential model search.

### Outcome measures

We will focus on two outcomes measures: estimates of numbers of incident and prevalent cases per 1,000 for the year 2007 and the ranking of the 18 diseases in terms of incidence and prevalence. Disease specific predictions from the regression models specified by age and sex and their fit to the data are only presented for diabetes in the text. Predictions of the fitted regression models for incidence and prevalence were weighted by the age and sex distribution of the Dutch population as of 1/1/2007 and presented as cases per 1,000.

To estimate cases per 1,000 and the associated uncertainty, we made two different types of predictions using the model. First, we used predictions of the regression models for the 'average' GP registration, that is by fixing the 'random' intercept at its average value on the incidence or prevalence scale (which is different from setting the random intercept at zero in case of the log link [25]). We only took into account uncertainty in the parameters of the "fixed effects" of the regression models (thus, only the uncertainty surrounding the coefficients used to model age and gender). This way, we estimated confidence intervals taking as reference the 'average' registration. Secondly, we also took into account the variance surrounding the intercept, i.e. the "random effect". This provides insight into the uncertainty that is due to differences between GPRNs.

A stochastic ranking (a ranking taking into account uncertainty) was computed of the incidence and prevalence of diseases using Monte Carlo simulations. Regression coefficients of the regression models for the different diseases were repeatedly drawn from a multivariate normal distribution on the link scale including the between GPRN variance. For each draw of the regression coefficients and random intercepts, incidence and prevalence numbers were calculated for all diseases. Subsequently, for each draw the diseases were ranked in descending order of magnitude for both incidence and prevalence. Sample size for the Monte Carlo simulation was set at 5,000 resulting in 5,000 rankings. This allowed us to compute what rankings of diseases were most likely and to what extent shifts in ranks may occur due to uncertainty in the model parameters. Sample size for the simulation was set at 5,000 as this sample size resulted in an accurate replication of the confidence intervals obtained from the regression models.

## Results

### Regression models

**Figure 1:**
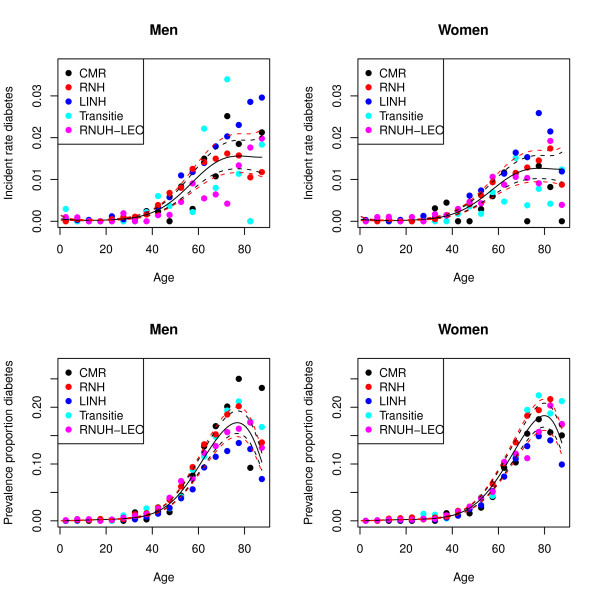
**Incidence and prevalence for diabetes**. Dots indicate the raw from the different GPRN's, lines indicate predictions from the regression models. The black broken lines indicate prediction intervals using the fixed effects estimates only while the red lines broken lines include the between GPRN variance.

To illustrate the age and sex specific estimates obtained with the regression models Figure 1 displays data of diabetes incidence and prevalence for the different GPRNs and predictions of the regression models. The regression model for diabetes incidence contained 4 terms for age (age, age^2, age^3 and age^4) and one dummy variable for gender. Figure 1 illustrates the effect of the variation between GPRNs and this is reflected by the fact that confidence intervals around the model predictions are wider if the variance surrounding the random intercept is included.

Table 2 displays a summary of the variables included in the 'optimal' regression models according to the Bayesian Information Criterion. For instance, the optimal model for heart failure prevalence include the variables age, age squared, age cubed, sex and a term for the interaction between sex and age.

**Table 2:** parameters in fitted regression models

Disease name	Incidence	Prevalence
Diabetes	*f(age, age^2,age^3,age^4,sex)*	f(age, age^2,age^3,age^4,age^5,age^6,sex,sex*age, sex*age^2)

Heart failure	f(age, age^2,sex,sex*age, sex*age^2)	f(age, age^2,age^3,sex,sex*age)

COPD	f(age, age^2,age^3,age^4,sex,sex*age, sex*age^2)	f(age, age^2,age^3,age^4, age^5, age^6,age^7,age^8, sex,sex*age, sex*age^2 sex*age^3 sex*age^4)

Stroke	*f(age, sex)*	*f(age, age^2,age^3,sex)*

Coronary heart disease	f(age, age^2,sex,sex*age, sex*age^2,sex*age^3)	f(age, age^2,sex,sex*age, sex*age^2)

Dementia	*f(age, age^2,age^3,sex)*	*f(age, age^2,age^3,sex)*

Schizophrenia	*f(age, age^2,sex)*	f(age, age^2,age^3,sex,sex*age)

Parkinson	*f(age, age^2,sex)*	*f(age, age^2,age^3,age^4,sex)*

Multiple sclerosis	*f(age, age^2,sex)*	*f(age, age^2,sex)*

Epilepsy	*f(age, age^2,sex)*	*f(age, age^2,age^3,age^4,age^5,sex)*

Macular degeneration	*f(age,sex)*	*f(age,sex)*

Glaucoma	*f(age, age^2,sex)*	*f(age, age^2,age^3,sex)*

Cataracts	*f(age, age^2,age^3,sex)*	*f(age, age^2,age^3,age^4,sex)*

Hearing impairments	*f(age, age^2,age^3,sex)*	f(age, age^2,age^3,age^4,age^5,sex,sex*age)

Inflammatory bowel diseases	*f(age, age^2,age^3,sex)*	*f(age, age^2,age^3,age^4,sex)*

Osteoporosis	f(age, sex,sex*age, sex*age^2)	f(age, sex,sex*age, sex*age^2,sex*age^3)

Rheumatoid arthritis	*f(age, age^2,sex)*	f(age, age^2,sex,sex*age)

Arthritis	*f(age, age^2,sex)*	f(age, age^2,sex,sex*age,age^2, sex*age^2,sex*age^3,sex*age^4)

### Population incidence and prevalence

**Figure 2:**
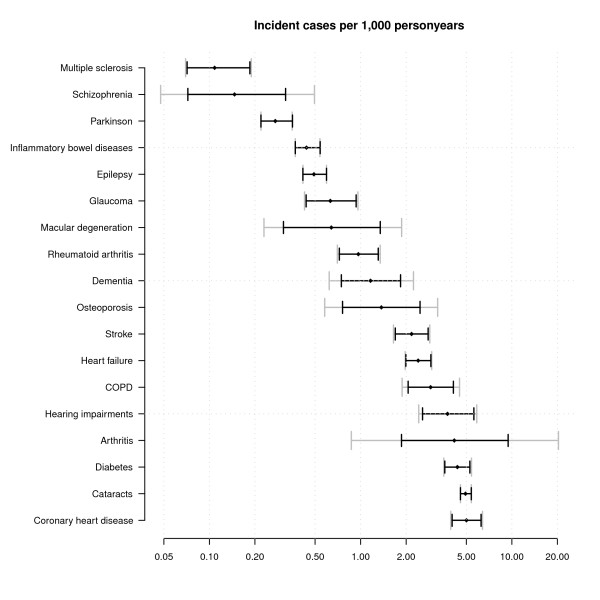
**Incident cases per 1,000 (men plus women) in the Netherlands 2007**. The black lines indicate prediction intervals using the fixed effects estimates only while the gray lines broken lines include the between GPRN variance.

**Figure 3:**
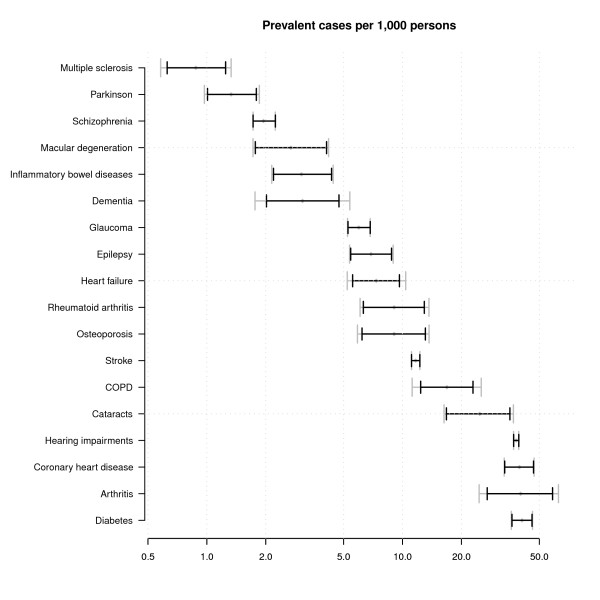
**Prevalence per 1,000 (men plus women) in the Netherlands 1 January 2007**. The black lines indicate prediction intervals using the fixed effects estimates only while the gray lines broken lines include the between GPRN variance.

Figures 2 and 3 display estimates of the numbers of incident and prevalent cases per 1,000 for 2007 together with 95% confidence intervals with and without the between GPRN variance (note that the x-axis is on the log scale in both figures and that in both Figure 2 and Figure 3 diseases are ranked by their point estimate). It can be seen from Figure 2 that coronary heart disease had the highest incidence in 2007 (more than 5 cases per 1000) and Multiple Sclerosis (MS) the lowest (a bit more than 0.1 case per 1,000). Confidence intervals around point estimates for total incidence are especially wide for arthritis ranging from less than 1 case per 1000 to more than 10 cases per 1000 personyears. Figure 3 shows that diabetes had the highest prevalence and MS the lowest. Confidence intervals around total prevalence are especially wide for diabetes, arthritis, coronary heart disease, cataracts and COPD. What also can be seen from Figures 2 and 3 is the influence of the variance of the random intercept. For some diseases confidence intervals widen enormously when including this variance (especially arthritis), while for other diseases the random effects variance was estimated to be very low (e.g. diabetes prevalence). In general, the between GPRN variance is higher for incidence than prevalence.

### Stochastic ranking of incidence and prevalence

Table 3 shows the stochastic ranking of disease incidences, with in the first column the diseases in descending order according to the point estimates of their incidence. The grey shaded probabilities indicate the rank positions which each disease is most likely to occupy (i.e. the maximum value of each row). The bold faced numbers indicate which disease is most likely to take a certain rank position (i.e. the maximum value of each column). For instance, if we look at ranking according to the point estimates arthritis is ranked 4^th^. However, arthritis can take ranks between 1 and 12 with the first rank being most probably. The disease that is most likely to take the first position in terms of incidence is arthritis. Arthritis, coronary heart disease, cataracts, diabetes and hearing impairments all have a non-zero probability to rank first in terms of incidence, although the probability for hearing impairments is below the 5% level.

**Table 3:** stochastic ranking of diseases in terms of incidence in 2007

	Probability to rank																	
	**1**	**2**	**3**	**4**	**5**	**6**	**7**	**8**	**9**	**10**	**11**	**12**	**13**	**14**	**15**	**16**	**17**	**18**

1 Coronary heart disease	0.30	0.34	0.22	0.11	0.03													

2 Cataracts	0.22	**0.43**	0.28	0.07	0.01													

3 Diabetes	0.04	0.11	**0.33**	**0.37**	0.14	0.01												

4 Arthritis	**0.38**	0.04	0.05	0.08	0.11	0.08	0.05	0.08	0.06	0.03	0.02	0.01						

5 Hearing impairments	0.05	0.07	0.11	0.28	**0.34**	0.12	0.02	0.01										

6 COPD		0.01	0.02	0.09	0.27	**0.38**	0.14	0.08	0.02									

7 Heart failure					0.05	0.25	**0.44**	0.21	0.03									

8 Stroke					0.02	0.11	0.28	**0.45**	0.13	0.01								

9 Osteoporosis				0.01	0.02	0.04	0.05	0.10	**0.35**	0.21	0.13	0.07	0.02	0.01				

10 Dementia						0.01	0.01	0.05	0.28	**0.33**	0.20	0.09	0.02	0.00				

11 Rheumatoid arthritis									0.08	0.30	**0.44**	0.16	0.02	0.00				

12 Macular degeneration								0.01	0.06	0.10	0.12	0.22	0.17	0.08	0.16	0.06	0.01	

13 Glaucoma										0.02	0.09	**0.40**	**0.37**	0.08	0.04	0.00		

14 Epilepsy												0.05	0.30	**0.49**	0.15	0.01		

15 Inflammatory bowel diseases												0.01	0.08	0.31	**0.57**	0.03		

16 Parkinson															0.05	**0.79**	0.15	

17 Schizophrenia												0.01	0.01	0.01	0.03	0.11	**0.50**	0.33

18 Multiple sclerosis																	0.33	**0.67**

Diseases are ordered by rank as obtained by point estimates: bold face reflects the disease that is most likely to be at that position (i.e. the maximum value of each column); grey shade reflects at which position the disease is most likely to be ranked (i.e. the maximum value of each row).

Table 4 shows the stochastic ranking of disease prevalence. Arthritis has the highest probability of being first in rank while diabetes is highest in rank if we look at point estimates. This is due to the fact that the point estimates of both diseases are very close to each other with their confidence intervals largely overlapping. The ranking of disease prevalence is similar to the ranking of disease incidence. Differences in the incidence and prevalence ranking can partly be explained by differences in lethality of the disease. For example, coronary heart is, due to its detrimental effects on life expectancy, ranked lower in terms prevalence than in terms of incidence. From both the ranking of incidence and prevalence we can see that if uncertainty margins overlap the uncertainty interval of one disease may influence the stochastic ranking of other diseases.

**Table 4:** stochastic ranking of diseases in terms of prevalence 1/1/2007

	Probability to rank																	
	**1**	**2**	**3**	**4**	**5**	**6**	**7**	**8**	**9**	**10**	**11**	**12**	**13**	**14**	**15**	**16**	**17**	**18**

1 Diabetes	0.34	**0.40**	0.19	0.07														

2 Arthritis	**0.42**	0.12	0.09	0.32	0.06													

3 Coronary heart disease	0.22	0.30	0.26	0.21	0.01													

4 Hearing impairments	0.02	0.18	**0.44**	**0.34**	0.01													

5 Cataracts		0.01	0.01	0.06	**0.82**	0.10	0.00											

6 COPD					0.10	**0.85**	0.04	0.01										

7 Stroke						0.03	**0.75**	0.20	0.02									

8 Osteoporosis						0.01	0.10	0.34	0.32	0.14	0.06	0.03						

9 Rheumatoid arthritis						0.01	0.10	**0.34**	**0.33**	0.13	0.06	0.02						

10 Heart failure								0.08	0.21	**0.35**	0.24	0.12						

11 Epilepsy								0.03	0.12	0.33	**0.38**	0.14						

12 Glaucoma										0.05	0.26	**0.68**	0.01					

13 Dementia												0.01	**0.41**	0.29	0.22	0.05	0.01	

14 Inflammatory bowel													0.39	**0.40**	0.20	0.01		

15 Macular degeneration													0.18	0.30	**0.43**	0.08	0.01	

16 Schizophrenia														0.01	0.15	**0.83**	0.01	

17 Parkinson																0.02	**0.92**	0.06

18 Multiple sclerosis																	0.06	**0.94**

Diseases are ordered by rank as obtained by point estimates: bold face reflects the disease that is most likely to be at that position (i.e. the maximum value of each column): grey shade reflects at which position the disease is most likely to be ranked (i.e. the maximum value of each row).

## Discussion and conclusions

In this study we showed how routinely collected data from several general practitioners registration networks can be combined to estimate incidence and prevalence for 18 chronic diseases. Uncertainty around estimates of incidence and prevalence was looked at from different angles. First, we estimated confidence intervals both with and without the between GPRN variance. Next, we calculated a stochastic ranking of diseases in terms of incidences and prevalences. This was done using Monte Carlo simulation, which consisted of randomly drawing from the estimated probability distributions of the disease prevalences and incidences, each time obtaining a particular ordering. After repeating this process many times, a distribution of rankings emerges that provides insight into the likelihood of orderings different from the "standard" ordering based on the point estimates. Our simulations showed that the presence of wide confidence intervals for some individual diseases also influences the rank of other diseases with smaller confidence intervals. In this respect, the results of our study showed that league tables of diseases should be interpreted with caution as the standard (point estimates) ordering may not reflect the "real" ordering accurately. These findings obviously have relevance for public health policy and the monitoring of chronic diseases. Furthermore, our findings beg the need for effective tools to communicate uncertainty to policy makers.

It should be noted that in this study we only presented a ranking of a small set of diseases. Increasing the number of diseases would probably imply more overlap in confidence intervals and, therefore, more uncertainty in the ranking. Furthermore, in constructing the stochastic ranking we assumed independence between diseases. However, some diseases are causally related (e.g. diabetes and coronary heart disease) or share common risk factors such as smoking and body mass index. If the dependency between diseases could be quantified the stochastic ranking of diseases might again turn out to be different.

A drawback of our study is that we only used secondary data at the GPRN level that did not include demographic characteristics besides age and sex and that we could not assess disease classifications errors within our data set [26-28]. We did not have data at the GP practice level and could not verify how accurately GP's establish their diagnoses and code them into their computerised files. We can only hope that they maintain their professional standards and adhere to the professional standards as set forth in guidelines. Given these limitations regarding the crude data of our study, we employed methods of analysis that were chosen to take these potential sources of bias into account. The major challenge was to combine the data while doing justice to the heterogeneity between the different registries. This was achieved by modelling the data in a hierarchical/multi-level fashion using generalized linear models, in which the registries were modelled as a random intercept. Since the goal of our study was explicitly to focus on the typical (in our case average) GPRN instead of average subject we have chosen a random effects model over a GEE model [29]. This assumes that there is such thing as an "average" practice, which can be used to extrapolate estimates valid for the population. An additional advantage of the random effects model is that it provided an estimate of the variability between GPRNs. We have presented this variability graphically and have incorporated it in our simulations and saw that especially for incidence the between GPRN variance was considerable. This could suggest that GPRNs might differ in the distinction between 'new' and 'old' cases.

There are several ways to estimate morbidity rates in a population in a cross-sectional manner [28]. Examples are health interview surveys and health examination surveys. It is obvious that health interview surveys have inherent limitations as they rely on self-report. Hospital-based data, although readily available in many countries, have the important disadvantage of selection bias as these include only those cases that lead to hospitalization. Information gathered in general practices, on the other hand, does not have these drawbacks. In countries in which the majority of the population is registered with a GP, such as the UK and the Netherlands, a source is available that could, in principle, be used to derive reliable, and representative estimates of descriptors of national health [30-32]. In the best of all possible worlds, all GPs would diagnose and document disease (episodes) in a uniform manner, reporting them comprehensively to a central databank. However, in reality there are important obstacles to drawing from this well in a sound manner. Firstly, there is the limited availability of data, because a national system of data collection for primary health care does not exist in most countries. Fortunately, several GPRNs have been started in the Netherlands over the past decades, which collect and share their morbidity data on a regular and structured basis. Secondly, diagnostic criteria and procedures are not always clear-cut and unambiguous, leaving room for differences in "case finding" and case ascertainment [28]. Under such conditions, differences between GPRNs (as captured in our models by the random intercept) can lead to big differences in estimates of prevalence and incidence. We hypothesize that for diseases for which uncertainty intervals are wide (such as arthritis), differences in case finding between GPRNs may offer an explanation for our findings [28]. Furthermore, differences in registration length between GPRNs might explain the between GPRN variance for some diseases because the longer the registration period the lower the probability that a prevalent case is misclassified as incident [33].

Concluding, estimates of incidence and prevalence can be obtained by combining data from different GPRNs, but confidence intervals must be considered. Monte Carlo simulation techniques can be utilized to assess uncertainty in the relative rankings of diseases. League tables of diseases based on point estimates should be interpreted with caution.

## Competing interests

The authors declare that they have no competing interests.

## Authors' contributions

PB did the analyses and drafted the initial manuscript. RH and HB contributed to the development of the methodology. All authors helped interpret the results and contributed to the writing of the manuscript.

## Pre-publication history

The pre-publication history for this paper can be accessed here:

http://www.biomedcentral.com/1471-2458/11/163/prepub
